# Relationship among symptoms, quality of life, and complementary and alternative medicines use among patients undergoing hemodialysis in French-speaking Switzerland

**DOI:** 10.1186/s12906-023-04001-z

**Published:** 2023-05-31

**Authors:** Marie Kemnitz, Matteo Antonini, Philippe Delmas

**Affiliations:** 1 University of Lausanne, Lausanne, Switzerland; 2 La Source School of Nursing, University of Applied Arts and Sciences Western Switzerland - HES-SO, Lausanne, Switzerland

**Keywords:** Hemodialysis, Quality of Life, Symptoms

## Abstract

**Background:**

Chronic renal disease is considered a main public health problem due to its high prevalence in the population. The solution of choice currently available is kidney transplantation but when this option is not available, blood purification treatments, notably haemodialysis (HD), are necessary. The presence of chronic renal disease combined with this demanding medical procedure leads to a hard symptomatology. To face this situation, HD patients often resort to complementary and alternative medicines (CAM) as they perceive that the healthcare professionals aren’t paying enough attention to their quality of life.

Given this background, we aim to describe the prevalence and the type of the CAM used among HD patients and their possible relations with patients’ symptomatology and quality of life.

**Methods:**

We interviewed 88 patients, undergoing hemodialysis in three hemodialysis centers in French-speaking Switzerland, about the presence of symptoms, their quality of life, and the possible use of CAM. Cluster analysis was used to create patients’ profiles about CAM use and regression analysis to explore the links between symptoms’ presence, patients’ quality of life, and CAM use.

**Results:**

Our results show a large use of CAM: almost two HD patients out of three uses at least one CAM. Using cluster analysis, we were able to identify five patients’ profiles: non-users (37.5% of our sample), users of herbal medicine (20.5%), users of prayer-based practices (18.2%), people mainly using massages (9.1%), and a residual group including the users of other CAMs, with a predominance of meditation (14.8%). As expected, we observe a negative relation between the number of declared symptoms and patients' quality of life. Contrarily, we observe no relation between the use of CAM and the presence of symptoms. Our results show a positive relationship between the use of CAM and patients’ overall perception of health as well as the psychological dimension of their quality of life. No relationship is observed with other dimensions of quality of life, notably the physical dimension.

**Conclusions:**

Our results suggests that CAMs aren’t used as a substitute of official medicine but as a parallel support to HD patients’ quality of life.

**Supplementary Information:**

The online version contains supplementary material available at 10.1186/s12906-023-04001-z.

## Background

Chronic renal disease is now considered a main public health problem due to its high prevalence in the population of many western countries [1]. In Switzerland, the context of our study, 350,000 people were suffering from chronic renal disease in 2020, and 30,000 were affected by severe or end stage renal disease (ESRD) [2, 3]. Patients suffering from ESRD need intensive medical support to prevent death [4].The solution of choice is kidney transplantation but, when this option is not available or during the waiting time before transplantation, blood purification treatments are necessary. In Switzerland, the most common treatment is haemodialysis (HD) [2, 3], an option that 4,704 people chose in 2019 [5]. HD is a demanding procedure consisting in pumping the blood through a special filter, called a dialyzer, and pumping the filtered blood back into the body.

HD patients are required to complete the four-hour procedure three times a week, usually in a hospital HD unit [6]. HD is, therefore, a quite invasive procedure that requires HD patients’ time, physical and psychological energies, and prevents them from taking part in many social and work-related activities. Moreover, HD demands a strict lifestyle including, but not limited to, the regular consumption of medicines, strict dietary rules, and a sharp limitation in the consumption of liquids [7]. Given these characteristics, the start of HD is often described as a real transition in patients’ lives [8].

The presence of ESRD, often associated with other chronic disease, combined with a demanding medical procedure leads to a hard symptomatology in HD patients [9, 10]. Almutary’s systematic review shows an average prevalence of symptoms ranging from 6 to 20, with lack of energy, sleepiness, and pain being the most prevalent [11]. Swiss data appear in line with these results with an average of 10 reported symptoms, the most prevalent being lack of energy, dry skin, trouble falling asleep, trouble staying asleep, and muscle cramps [12]. Ultimately, the heavy presence of symptoms and the constraints imposed by HD have a negative impact on HD patients’ quality of life (QoL) [13]. This aspect is sometimes neglected by healthcare professionals due to the traditional, and still present, lack of attention by the medicine toward everything that goes beyond the mere patients’ physical condition, the lack of holistic vision in favor of a symptom-based plan [14–16], and the degradation in the relationship between HD patients and healthcare professionals, in particular HD nurses, that is observed in some contexts [17–20]. Consequentially, HD patients look for alternative ways to cope with their symptoms and support their QoL, one of them being the use complementary and alternative medicines (CAM) [21]. Their use among HD patient is widely documented in recent literature with utilization rates that span between 26 and 65% [21–35]. The most used CAM appear to be psychotherapy [21, 22, 25, 27, 28, 31, 33], body-mind practices such as meditation, [21–23, 27, 28, 32], and spiritual practices [24, 27, 28]. The effects of CAM on symptoms are still debated and largely depend on the type of CAM used by HD patients, although effects on anxiety [36] and even mortality rates [37] were reported. The appeal of CAM is not without its risks, especially when HD patients don’t inform healthcare professionals about the practices they use; a wide-spread behavior [21, 22, 24, 25, 27–29, 31, 34]. Putting aside the previously described degraded relationship that affects some HD units, this lack of communication is due to both a certain reticence by HD patients to speak of something that they consider disconnected from medical practices and to a lack of interest and/or competence by healthcare professionals to frame HD patients’ CAM use [22, 27, 29]. This lack of communication can become a main issue for the efficacy of treatments and HD patients’ safety [21] as the use of CAM may interfere with usual treatments or having other side effects, notably but not limited to hyperglycemia, hypokalemia, or low blood pressure [21, 22, 30, 31].

Given this background, a central role in HD patients’ safety and QoL is played by healthcare professionals’ capacity to frame CAM use and the relation between HD patients’ and healthcare professionals. This is necessary to avoid unwanted consequences of CAM use and support HD patients’ QoL, irrespective of healthcare professionals’ personal opinions on CAM use. Healthcare professionals’ capacity to frame CAM use must be rooted in scientific data on CAM use among HD patients and they role they have in HD patients’ healthcare strategies. As CAM use often differs according to cultural contexts (see [38] or [39] for a review of studies in different countries); multiple studies in different areas are needed both to find general trends, and local specificities that must be considered by healthcare professionals.

In line with this perspective, our research aims to contribute to the description of the presence and the type of the CAM used among HD patients and their possible relations with patients’ symptomatology and QoL, in the context of French-speaking Switzerland.

## Methods

Our research has a twofold objective: first, we aim to describe the presence and the type of CAM used by HD patients in three HD units in French-speaking Switzerland. Second, we aim to estimate if the use of CAM is related to HD patients’ perception of symptoms and/or quality of life.

Our study is structured around a descriptive correlational research design. Data were collected between February and March 2022 in Clinique Cecil (Lausanne) Ensemble Hospitalier de la Côte (EHC, Morges) and Fribourg Hospital (HFR, Fribourg) all situated in French-speaking Switzerland. The target population included 173 patients. The following inclusion criteria were applied to create our sample: 1) have been continuously under hemodialysis for at least 6 months, 2) being 18 years or older, 3) being able to read and understand French, 4) being able to provide informed consent, 5) willingness to participate. In addition, the following non-inclusion criteria were applied: 1) suffering from diagnosed mental troubles, 2) being hospitalized for severe complications during the recruitment period. Power analysis conducted before data collection indicated a minimal sample size of 86 patients given a significance threshold of 5%, a power of 0.8, and a minimum correlation of 0.3, which is usually considered the lower limit for the score of a medium correlation. After data collection, the final sample size included 88 HD patients. All the HD patients were personally met by the researchers to collect their informed consent. Patients completed the questionnaire on paper during their dialysis sessions and researchers were always present to help patients complete the questionnaire.

Data was collected using a four-part questionnaire. The first part served to collect sociodemographic and health-related data. Questions covered gender, age, marital status, activity status, presence of children, smoking status, months in hemodialysis, the use of medical treatments, and comorbidities. This part of the questionnaire was successfully used in the past [40].

The second part includes the French version [41] of the « Dialysis Symptom Index» (DSI) by Weisbord et al. [42] which includes a list of 30 self-perceived symptoms whose presence and intensity is measured. This inventory is largely used internationally [10, 43–47] and has shown satisfying psychometric features [42]. For the scope of this research, we use only the presence/absence of symptoms, excluding the perceived intensity of them.

The third part of the questionnaire consisted of the « World Health Organization Quality of Life Bref» (WHOQOL-BREF) in its French version [48]. This scale includes two questions, on general QoL and general health, and four dimensions on self-perceived physical (7 items), psychological (6 items), social (3 items) and environmental (8 items) QoL. This instrument has shown satisfying psychometric qualities [48]. The choice of this instrument has two main advantages. First, it doesn’t directly take in account symptoms as determinants of QoL. As we measure symptoms with another instrument, choosing the WHOQOL-BREF allows us to avoid redundance. Second, WHOQOL-BREF different dimensions allow for a more detailed description of QoL having two main dimensions (general QoL and general health) and four sub-dimensions (physical, psychological, social, and environmental).

The fourth part of our questionnaire includes the French version of the «International-Complementary and Alternative Medicine questionnaire» (I-CAM-Q) [49, 50]. This instrument is composed by four sections: (a) meetings with medical and CAM practitioners (physician, homeopath, acupuncture practitioner, osteopath, chiropractor, aromatherapist), (b) the type of practices linked to CAM used and requiring the presence of a practitioner, (c) the products linked to CAM used, (d) the type of practices linked to CAM use that does not necessarily require the presence of a practitioner. Each section asks for the frequency of each specific behavior, the motivation behind its use, and the perceived efficacy. Moreover, the questionnaire allows the respondent to add further categories to complete the options given in the instruments. The I-CAM-Q was already used in surveys about CAM use, notably in the pan-European CAMbrella project [49, 50]. In this paper, we will focus solely on the use/non-use of CAM described in Sect. 2, i.e. the type of practices that requires the presence of a practitioner, and Sect. 4, i.e. the self-aid practices.

As for statistical analysis software R version 4.1.2 [51] was used. First, means, standard deviations, and frequencies were calculated for sociodemographic and health-related data, as well as for data on patients’ symptoms, QoL, and the use of CAM. Second, cluster analysis was used to create a typology of CAM users. The dichotomic variables indicating the use of each type of CAM are organized in sequence-like structures where the order of the variables does not matter. The matrix of distances is formed using simple Hamming distances [52] that allows only for substitutions, and a constant substitution matrix. Clustering follows the procedure introduced by Studer [53] and consists of a sequential combination of Ward’s and partition around medoids (PAM) clustering. To evaluate the adequate number of clusters, we used weighted average silhouette width analysis [54] and Hubert’s C Index [55]. Third, regression analysis was used to estimate the relations between (a) perceived symptom presence and perceived patients’ QoL, (b) CAM use and perceived symptom presence, (c) CAM use and perceived patients’ QoL. Patients’ sociodemographic and health characteristics were introduced in the models as control variables. Statistical significance for all tests was set at *p* < 0.05, following a frequentist approach, and no imputation were used for missing data.

Our research was approved by the Canton of Vaud Research Ethics Board pursuant to Swiss federal law respecting human research, ethical clearance was granted (2021–02023), and all methods were performed in accordance with the relevant guidelines and regulations.

## Results

After data collection, our sample was composed by 88 out of the 122 contacted HD patients with a participation rate of 72%. Main reasons for refusing the participation to our study were being too tired, or not being interested. The sociodemographic and clinical data of the patients in our sample are presented in Table 1.

**Table 1:** Sociodemographic and clinical data

		**mean (SD)**	**N**	**%**
**Age**		68.6 (13.1)		
**Sex**				
	Women		30	34.1
	Men		58	65.9
**Civil status**				
	Single		12	13.6
	Married		47	53.4
	Separated/divorced		15	17.1
	Free union		1	1.1
	Widow		13	14.8
**Activity status**				
	Inactive		18	20.4
	Working		10	11.4
	Retired		60	68.2
**Children**				
	Yes		31	35.2
	No		57	64.8
**Smoking status**				
	Former smoker		34	38.6
	Current smoker		10	11.4
	Non-smoker		44	50.0
**Months in hemodialysis**		45.0 (54.2)		
**Other methods of dialysis used**				
	Yes		9	10.2
	No		79	89.8
**On transplantation waiting list**				
	Yes		37	42.0
	No		48	54.6
**Medical treatments**				
	Antihypertensive		48	54.6
	Vitamins		34	38.6
	Iron supplements		23	26.1
	Painkiller		39	44.3
	Antidepressants		11	12.5
	Others		39	44.3
**Comorbidities**				
	Cardiac insufficiency		11	12.5
	Arterial hypertension		49	55.7
	Diabetes		35	39.8
	Chronic obstructive pulmonary disease		5	5.7
	Heart arrhythmia		16	18.2
	Legs arteritis		11	12.5
	Cancer		11	12.5
	Hepatitis		4	4.6

Our results are in line with previous data on this population [12, 56, 57]: men represent the majority of respondent (65.9%). The average age is quite high reaching 69 years (SD = 13.1), consequently the majority of respondents (68.2%) are already retired. Active people are only a small minority of respondents (11.4%). As for the clinical situation, patients were under HD for an average of 45 months (SD = 54.2), only a small minority has previously used other form of treatment (10.2%), and less than half are on the transplantation waiting list (42.0%). Because of HD patients’ health conditions, the use of medical treatments is very common, in particular antihypertensives (54.6%), painkillers (44.3%), vitamins (38.6%), iron supplements (26.1%), and antidepressants (12.5%). Comorbidities were also widespread, specifically hypertension (55.7%) and diabetes (39.8%), both often related with ESRD.

Thirty symptoms typically associated with ESRD and HD [42] were explored (see Table 2) and the patients showed an average presence of 8.9 symptoms (SD = 4.6), in line with previous studies on the same population [12, 57]. The most frequently observed are tiredness and lack of energy (63.6%), dry skin (58.0%), trouble staying asleep (45.5%), trouble falling asleep (44.3%), and shortness of breath (44.3%).

**Table 2:** Symptoms presence and intensity

Symptoms	N (%)
Chest pain	2 (2.3%)
Vomiting	8 (9.0%)
Numbness or tingling in feet	13 (14.8%)
Nausea	13 (14.8%)
Difficulty concentrating	13 (14.8%)
Lightheadedness or dizziness	14 (15.9%)
Diarrhea	15 (17.0%)
Headache	16 (18.2%)
Decreased appetite	17 (19.3%)
Constipation	18 (20.5%)
Swelling in legs	18 (20.5%)
Cough	20 (22.7%)
Feeling irritable	23 (26.1%)
Feeling anxious	25 (28.4%)
Muscle soreness	26 (29.6%)
Feeling sad	28 (31.8%)
Numbness or tingling in feet	29 (33.0%)
Itching	29 (33.0%)
Feeling nervous	29 (33.0%)
Worrying	30 (34.0%)
Dry mouth	32 (36.4%)
Muscle cramps	35 (39.8%)
Bone or joint pain	35 (39.8%)
Decreased interest in sex	35 (39.8%)
Difficulty becoming sexually aroused	37 (42.0%)
Shortness of breath	39 (44.3%)
Trouble falling asleep	39 (44.3%)
Trouble staying asleep	40 (45.5%)
Dry skin	51 (58.0%)
Feeling tired or lack of energy	56 (63.6%)
Average presence of symptoms	8.9 (SD = 4.6)

HD patients’ QoL was initially described by two overall dimensions: overall quality of life and overall perceived health. The former scores a fairly good level, 3.7 (SD = 0.9) on a 1 to 5 Likert scale, the latter a moderate level, i.e. 3.0 (SD = 1.2). As for the four dimensions of HD patients’ QoL, the physical dimension of QoL, which is described as a person’s physical condition that influences their quality of life, has the lowest score: 3.3 (SD = 0.8). The second lowest score, i.e. 3.9 (SD = 0.9), was recorded by the social dimension, which describes the impact of people network on HD patients’ QoL. The psychological dimension, which is described as a person’s psychological balance that influences their quality of life, received a slightly higher score of HD patients’ QoL (4.1, SD = 0.7). Finally, the highest value is linked to the environmental dimension: 4.5 (SD = 0.5). This dimension describes how the surrounding natural environment and housing conditions influence people’s QoL.

Lastly, patients’ use of CAM is described in Table 3. More than half of the interviewed HD patients have used at least one CAM (63.6%). The most used CAMs were prayer (27.3%), herbal medicine (20.5%), and meditation (13.6%).

**Table 3:** CAM type and frequency

	**N**	**%**
Homeopathy _(CAM1)_	8	9.1
Acupuncture _(CAM2)_	6	6.8
Vertebral manipulation _(CAM3)_	1	1.1
Osteopathy _(CAM4)_	7	8.0
Mesotherapy _(CAM5)_	0	0.0
Herbal medicine _(CAM6)_	14	15.9
Massages _(CAM7)_	9	10.2
Meditation _(CAM8)_	12	13.6
Yoga _(CAM9)_	1	1.1
Qi gong _(CAM10)_	0	0.0
Taiichi _(CAM11)_	0	0.0
Sophrology _(CAM12)_	4	4.6
Pray _(CAM13)_	24	27.3
Physical activity _(CAM14)_	5	5.7
Other treatments that need the presence of a practitioner	16	18.2
Other treatments that don’t need the presence of a practitioner	6	6.8
People using at least a CAM	56	63.3

**Fig. 1:**
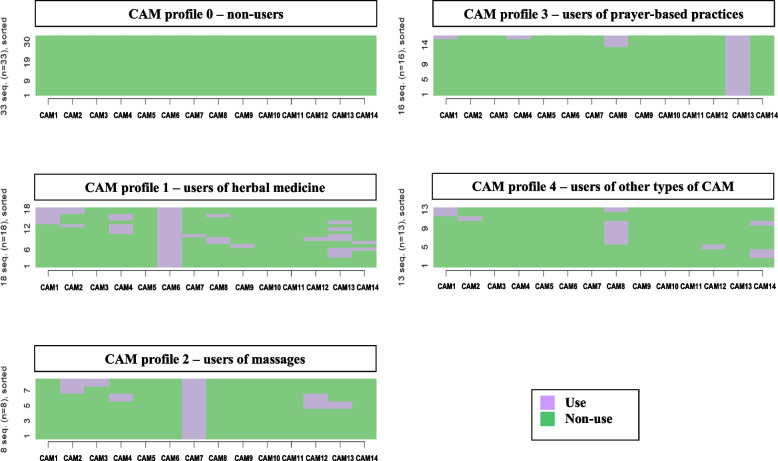
CAM users’ profiles

With the collected data on CAMs used, we were able to define 5 groups of CAMs users (see Fig. 1). Each group is represented in a graph. The graphs can be read similarly to a table as each line represents a patient, and each column indicates a specific CAM. The overall view of each graph shows if any CAM is used by all the patients in the cluster (i.e. a solid column of “use”), if it is used but only by some of the patients in the cluster (i.e. a column with both “use” and “non-use”), or if it is used by none of the patients in the cluster (i.e. a solid column of “non-use”).

The first group (CAM profile 0) was manually defined and include all the patients that don’t use any CAM (37,5% of the patients in our sample). The other groups were defined using cluster analysis. The group “CAM profile 1” describes people using herbal medicine _(CAM6)_ (20,5% of our sample). As shown in the Fig. 1, all the patients in this cluster use the herbal medicine _(CAM6)_ and often other CAMs, in particular, prayer-based practices _(CAM13)_, homeopathy _(CAM1)_, and osteopathy _(CAM4)_. The “CAM profile 2” group includes the patients that use massages _(CAM7)_ as a CAM (9,1% of our sample). In this group, other CAMs are less present, even if we observe the presence of a few other practices, notably acupuncture _(CAM2)_ and sophrology _(CAM12)_. The group “CAM profile 3” describes people who use prayer-based practices _(CAM13)_ (18,2% of our sample). As it shown by the almost complete absence of “use” referred to other CAMs (see Fig. 1), the patients in this cluster almost never use other CAMs, except for a small presence of meditation _(CAM8)_. The use of meditation _(CAM8)_ may be connected to the main practice in this group, i.e. the use prayer-based practices _(CAM13)_, as meditation recalls some forms of prayer. Finally, the “CAM profile 4” group includes patients’ using the other types of CAM, in particular meditation _(CAM8)_ (14,8% of our sample). Nevertheless, no CAM is used by all the patients included in this cluster.

The relations between HD patients’ symptoms, CAMs use, and QoL were described using regression analysis. To describe patients’ symptoms, we considered the number of symptoms reported. As for CAMs use, it is described in two ways. First, we simply divide users and non-users, and we compared these two groups. Second, we used the just defined typology of CAM users (see Fig. 1) to test if every profile is equally connected with patients’ QoL. Finally, to describe patients’ QoL, we use the scores associated to the “overall health” question and the scores of two dimensions: “psychological QoL” and “physical QoL”. We limited the analysis to these dimensions as we expect them to be directly impacted by both patients’ symptoms and CAM use. Even if they may be indirectly affected, we decide to exclude from our main analyses the scores referred to the question about general levels of QoL, and the “environmental” and “social” dimensions as, given the way these scales are constructed, they are more closely affected by other aspects of patients’ lives, such as their social network and housing facilities, in comparison to patients’ symptoms or CAM use.^1^

Table 4 summarizes the relation between symptoms and CAM use. Both using the simple dichotomous variable user/non-user (model 1) and the CAM users’ profiles (model 2), we observe no relation between CAM use and the number of declared symptoms. As a sensitivity analysis [58] we have inverted the dependent and the independent variables. Still no significant relation is observed.

**Table 4:** Relation between CAM use and symptoms

	**Model 1**		**Model 2**	
	**Beta**	***p*-value**	**Beta**	***p*-value**
**Intercept**	-0.53	0.867	7.96	0.106
**CAM use, yes**	0.14	0.082	xxx	xxx
**CAM profile 1**	xxx	xxx	2.85	0.112
**CAM profile 2**	xxx	xxx	0.05	0.979
**CAM profile 3**	xxx	xxx	1.16	0.402
**CAM profile 4**	xxx	xxx	1.89	0.219
**Age, years**	-0.02	0.694	-0.04	0.550
**Gender, male**	-0.93	0.210	-1.11	0.333
**Civil status, separated**	-0.24	0.836	2.57	0.187
**Civil status, free union**	0.02	0.985	2.27	0.220
**Civil status, widow**	0.60	0.641	2.55	0.266
**Work status, inactive**	0.46	0.746	-2.28	0.302
**Work status, retired**	-0.02	0.983	-3.32	0.072
**Children, yes**	0.30	0.693	-0.23	0.856
**Smoking status, old smoker**	-0.33	0.681	0.19	0.875
**Smoking status, current smoker**	-1.09	0.357	-0.27	0.886
**Months in hemodialysis**	≈0.00	0.846	0.01	0.355
**Other method of dialysis, yes**	1.37	0.289	-0.21	0.916
**On transplantation waiting list, yes**	1.59	0.064	0.47	0.725
**Use of antihypertensive, yes**	0.78	0.485	-1.34	0.442
**Use of vitamins, yes**	-0.18	0.854	-2.28	0.141
**Use of iron complements, yes**	-0.53	0.512	1.42	0.332
**Use of painkiller, yes**	-0.41	0.573	3.30	0.004
**Use of antidepressants, yes**	2.00	0.127	3.55	0.021
**Use of other treatments, yes**	0.08	0.903	0.20	0.847
**Cardiac insufficiency, present**	-0.03	0.977	-0.82	0.619
**Arterial hypertension, present**	-0.64	0.573	2.07	0.210
**Diabetes, present**	0.58	0.370	0.88	0.409
**COPD, present**	0.71	0.637	4.26	0.051
**Heart arrhythmia, present**	0.54	0.560	0.72	0.599
**Legs arteritis, present**	0.48	0.642	0.86	0.616
**Cancer, present**	1.84	0.182	4.02	0.034
**Hepatitis, present**	1.91	0.212	1.86	0.459
	*N* = 85		*N* = 85	
	McFadden's R-squared = 0.25		Adjusted R-squared= 0.27	

As for the overall health status (Table 5), we observe a negative relation with patient’s symptoms (models 3 and 4). This is an expected result and is quantified as a decrement of 0.16 points (for both models 3 and 4), on a 1 to 5 QoL Likert scale, for each present symptom. Contrarily, CAM use, both taking into consideration the use/non-use (model 3) and the CAM users’ profiles (model 4), appear unrelated to patients’ overall health status.

**Table 5:** Relation between symptoms presence and CAM use, and HD patients’ QoL (overall health)

	**Model 3**		**Model 4**	
	**Beta**	***p*-value**	**Beta**	***p*-value**
**Intercept**	3.52	0.011	4.01	0.004
**Symptoms, number**	-0.16	< 0.001	-0.16	< 0.001
**CAM profile 1**	xxx	xxx	0.72	0.149
**CAM profile 2**	xxx	xxx	0.39	0.470
**CAM profile 3**	xxx	xxx	0.65	0.087
**CAM profile 4**	xxx	xxx	-0.27	0.517
**CAM use, yes**	0.38	0.199	xxx	xxx
**Age, years**	≈0.00	0.865	-0.01	0.774
**Gender, male**	0.48	0.139	0.51	0.108
**Civil status, separated**	0.08	0.885	-0.19	0.723
**Civil status, free union**	-0.25	0.601	-0.63	0.217
**Civil status, widow**	0.42	0.499	0.13	0.841
**Work status, inactive**	0.03	0.960	0.03	0.956
**Work status, retired**	0.06	0.897	0.19	0.711
**Children, yes**	0.30	0.380	0.49	0.159
**Smoking status, old smoker**	-0.32	0.339	-0.35	0.283
**Smoking status, current smoker**	0.32	0.539	0.31	0.544
**Months in hemodialysis**	≈0.00	0.749	≈0.00	0.665
**Other method of dialysis, yes**	-0.12	0.831	≈0.00	0.996
**On transplantation waiting list, yes**	-0.30	0.412	-0.45	0.224
**Use of antihypertensive, yes**	0.01	0.977	0.06	0.898
**Use of vitamins, yes**	-0.22	0.588	-0.17	0.695
**Use of iron complements, yes**	0.22	0.561	0.19	0.637
**Use of painkiller, yes**	0.01	0.967	-0.09	0.788
**Use of antidepressants, yes**	-0.22	0.602	-0.33	0.449
**Use of others treatments, yes**	-0.14	0.613	-0.14	0.613
**Cardiac insufficiency, present**	0.25	0.560	0.14	0.747
**Arterial hypertension, present**	0.32	0.468	0.42	0.356
**Diabetes, present**	-0.34	0.239	-0.20	0.484
**COPD, present**	1.08	0.077	1.19	0.068
**Heart arrhythmia, present**	0.26	0.474	0.27	0.474
**Legs arteritis, present**	0.14	0.763	0.33	0.484
**Cancer, present**	0.08	0.883	0.31	0.550
**Hepatitis, present**	-0.01	0.989	-0.07	0.920
	*N* = 85		*N* = 85	
	Adjusted R-squared = 0.21		Adjusted R-squared = 0.24	

We obtain similar results when analyzing the physical dimension of QoL (Table 6). As expected, symptoms are negatively related to patients’ physical QoL. Reductions are estimated at -0.11 points, on the 1 to 5 QoL Likert scale, for each symptom present (in both model 5 and 6). As for the use of CAM, the general use seems not to be linked to patients’ physical QoL (model 5) but still a specific profile of CAM use, the patients that use prayer-based practices (CAM profile 3), appear to have their physical QoL affected positively (see model 6). People that use prayer-based practices have a physical QoL that is, on average, 0.47 points higher than the people using no CAM.

**Table 6:** Relation between presence of symptoms and CAM use and HD patients’ QoL (physical dimension)

	**Model 5**		**Model 6**	
	**Beta**	***p*-value**	**Beta**	***p*-value**
**Intercept**	3.12	< 0.001	3.28	< 0.001
**Symptoms, number**	-0.11	< 0.001	-0.11	< 0.001
**CAM profile 1**	xxx	xxx	0.27	0.334
**CAM profile 2**	xxx	xxx	0.38	0.213
**CAM profile 3**	xxx	xxx	0.47	0.029
**CAM profile 4**	xxx	xxx	-0.01	0.956
**CAM use, yes**	0.30	0.070	xxx	xxx
**Age, years**	0.01	0.337	0.01	0.535
**Gender, male**	0.39	0.029	0.40	0.028
**Civil status, separated**	0.36	0.216	0.25	0.413
**Civil status, free union**	0.16	0.554	0.04	0.887
**Civil status, widow**	0.23	0.497	0.12	0.731
**Work status, inactive**	0.17	0.595	0.12	0.722
**Work status, retired**	-0.18	0.502	-0.15	0.598
**Children, yes**	0.18	0.328	0.27	0.164
**Smoking status, old smoker**	-0.02	0.893	-0.04	0.841
**Smoking status, current smoker**	-0.18	0.538	-0.14	0.618
**Months in hemodialysis**	≈0.00	0.845	≈0.00	0.967
**Other method of dialysis, yes**	0.35	0.241	0.44	0.155
**On transplantation waiting list, yes**	0.15	0.460	0.11	0.582
**Use of antihypertensive, yes**	-0.18	0.477	-0.08	0.758
**Use of vitamins, yes**	0.13	0.565	0.22	0.367
**Use of iron supplements, yes**	0.28	0.174	0.20	0.383
**Use of painkiller, yes**	-0.43	0.017	-0.48	0.011
**Use of antidepressants, yes**	0.12	0.615	0.06	0.800
**Use of others treatments, yes**	0.02	0.885	0.03	0.858
**Cardiac insufficiency, present**	-0.43	0.077	-0.53	0.038
**Arterial hypertension, present**	-0.04	0.858	-0.06	0.802
**Diabetes, present**	-0.11	0.486	-0.05	0.752
**COPD, present**	0.18	0.597	0.21	0.536
**Heart arrhythmia, present**	0.36	0.076	0.40	0.061
**Legs arteritis, present**	0.38	0.139	0.47	0.078
**Cancer, present**	-0.45	0.127	-0.35	0.242
**Hepatitis, present**	-0.06	0.881	-0.08	0.838
	*N* = 85		*N* = 85	
	Adjusted R-squared = 0.39		Adjusted R-squared = 0.39	

Finally, we estimate the relation between patients’ symptoms, CAM use, and the psychological dimension of QoL (Table 7). Results are clearer in this case. Not only symptoms are linked with psychological QoL (-0.08 points, on the 1 to 5 QoL Likert scale, for each symptom present) but also the relationship with CAM use presents significant values. The general use of CAM seems to be positively related to patients’ QoL (model 7): CAM users have a psychological QoL that is, on average, 0.57 points higher than who doesn’t use CAM once the effects of control variables have been removed. Moreover, if we analyze the users’ profiles (model 8), we note that the overall relationship is driven by the people using prayers (CAM profile 3) and the group that uses a vast array of CAM, but principally meditation, (CAM profile 4). Effects are estimated at, respectively, + 0.73 and + 0.62 points, compared to patients’ that don’t use any CAM.

**Table 7:** Relation between symptoms presence and CAM use and HD patients’ QoL (psychological dimension)

	**Model 7**		**Model 8**	
	**Beta**	***p*-value**	**Beta**	***p*-value**
**Intercept**	3.18	< 0.001	3.19	< 0.001
**Symptoms, number**	-0.08	< 0.001	-0.08	< 0.001
**CAM profile 1**	xxx	xxx	0.27	0.27
**CAM profile 2**	xxx	xxx	0.37	0.17
**CAM profile 3**	xxx	xxx	0.73	< 0.001
**CAM profile 4**	xxx	xxx	0.62	< 0.001
**CAM use, yes**	0.57	< 0.001	xxx	xxx
**Age, years**	0.01	0.129	0.01	0.119
**Gender, male**	0.16	0.297	0.13	0.384
**Civil status, separated**	-0.28	0.270	-0.20	0.447
**Civil status, free union**	-0.25	0.273	-0.14	0.562
**Civil status, widow**	-0.16	0.586	-0.09	0.762
**Work status, inactive**	0.22	0.436	0.06	0.841
**Work status, retired**	-0.06	0.805	-0.21	0.403
**Children, yes**	0.38	0.021	0.37	0.028
**Smoking status, old smoker**	-0.24	0.130	-0.22	0.165
**Smoking status, current smoker**	-0.56	0.026	-0.55	0.029
**Months in hemodialysis**	≈0.00	0.165	≈0.00	0.114
**Other method of dialysis, yes**	0.45	0.085	0.52	0.052
**On transplantation waiting list, yes**	0.24	0.179	0.27	0.13
**Use of antihypertensive, yes**	0.18	0.414	0.20	0.382
**Use of vitamins, yes**	0.06	0.774	0.14	0.503
**Use of iron complements, yes**	0.05	0.782	-0.08	0.683
**Use of painkiller, yes**	0.08	0.580	0.14	0.377
**Use of antidepressants, yes**	-0.15	0.465	-0.13	0.527
**Use of others treatments, yes**	-0.20	0.141	-0.18	0.190
**Cardiac insufficiency, present**	0.17	0.404	0.11	0.605
**Arterial hypertension, present**	0.23	0.266	0.17	0.438
**Diabetes, present**	-0.07	0.620	-0.09	0.520
**COPD, present**	0.31	0.290	0.41	0.164
**Heart arrhythmia, present**	-0.05	0.786	0.05	0.784
**Legs arteritis, present**	0.24	0.270	0.18	0.430
**Cancer, present**	0.23	0.360	0.18	0.479
**Hepatitis, present**	-0.26	0.438	-0.22	0.520
	*N* = 85		*N* = 85	
	Adjusted R-squared = 0.42		Adjusted R-squared = 0.42	

## Discussion

Our research has explored the relation among HD patients’ symptoms, CAM use, and quality of life in the context of three HD units in French-speaking Switzerland. Our sample has similar characteristics to other samples of HD patients in the same area [12, 57]. We observe a male-dominated population with a relatively advanced age, and long experience of HD. Comorbidities and the use of medication is widespread as well as symptoms, due both to patients’ chronic healthcare problems and the HD side effects.

As expected, and in line with a large share of the literature [59, 60], the presence of symptoms appears linked to patients’ QoL. The more the number of symptoms increases, the more patients’ QoL decreases referring to the overall QoL, the physical and psychological dimensions alike. The burden of any new symptom, regardless of its cause, introduces new constraints and the need for further adaptation by the patients. This inevitably impacts negatively on all dimensions of patients’ QoL.

Considering the use of CAMs, we have observed that 63.6% of patients used at least one CAM. This is a strong presence, in line with some previous studies [21, 22, 24, 31, 33, 34] but higher than others [23, 27–29, 32]. Apart from regional differences due to HD patient’s cultural background and access to both medical treatments and CAMs, this partial discrepancy can be explained considering that the definition of CAM is still discussed in the literature [30]. Therefore, the rates of use may vary depending on which practices each definition includes. We used quite a large definition (see the CAMbrella project [49, 50, 61]) that includes some practices that are sometimes excluded, notably, the use of prayer-based practices with the goal of improving one’s own health condition. This practice is used by the 18.2% of our sample, quite a large share, and is consistent with the presence of an older population, more linked to a traditional religious practice [62]. This inclusion of prayer-based CAM and other practices could inflate the rate of CAM use in our sample. This would result in higher values, in comparison to other studies, just for a methodological discrepancy.

Aside from the group of non-users (CAM profile 0, 37.5% of the patients in our sample), and the users of prayer-based practices (CAM profile 3, 18.2%), our analyses have showed other types of CAM users. The larger group of users is represented by users of herbal medicine (CAM profile 1, 20.5%). Herbal medicine is a nomenclature that incorporates many types of practices. Results in different settings may diverge and previous studies has showed an even larger use among HD patients [21, 22, 24, 26, 27, 33, 34, 63]. A further group includes people mainly using massages (CAM profile 2, 9.1%). Again, this is a large category that includes various practices that may or may not involve a spiritual element. Finally, the last group is a residual group including the users of other CAM, with a predominance of meditation (CAM profile 4, 14.8%). Again, this is in line with previous studies that indicate a strong presence of mind–body practices, which include but aren’t limited to meditation [22, 28, 32–34]. Considering an overall view on our descriptive findings, our results are in line with previous literature that stress the presence of both spiritual practices [24, 27, 28] and body-mind practices, such as meditation, [22, 23, 32, 33], among the most used practices. Nevertheless, these terms are quite large and regional differences may appear comparing studies with larger samples and a detailed CAM nomenclature.

As for the relations between symptoms’ presence and CAM use, and CAM use and HD patients’ QoL, our data shows outputs only partially in line with previous results from the literature. We observe a lack of relationship between the use of CAM and the number of symptoms. This is in contrast with some previous studies that link these two elements [64–66]. Nevertheless, if we consider all our results at once, we can draw an interpretation that can explain this lack of relation. In fact, we observe that CAM use appears disconnected from self-defined overall health and the physical dimension of QoL, but we do observe a positive relation between CAM use and the psychological dimension of QoL. Therefore, the use of CAM seems to not be a simple substitute for what patients perceive to be lacking treatments that palliate symptoms effects at the physical level. If the use of CAM was a simple complement to official medicine, when patients feel they are lacking solutions, we should observe both a relation between symptoms and CAM use, and a relation between CAM use and the physical dimension of patients’ QoL. Patients’ behavior would follow a simple pattern: patients suffer from the consequences of their symptoms, they estimate the official medicine doesn’t have solutions for them, they appeal to the CAM that best fits their situation, and they find relief of their symptoms with positive consequences on patients’ physical QoL. Our results aren’t consistent with that model and suggest a different solution. The use of CAM appears linked to a better QoL but at the psychological level, not a physical one. That seems to suggest that HD patients are using CAM to maintain better psychological balance. Under that perspective, CAM doesn’t need to be associated to either the number of symptoms a patient has, nor the physical dimension of HD patients QoL. In fact, CAM use seems to have a much larger scope. Their efficacy in the reduction of the symptom presence, with the consequent positive effect on the physical dimension of QoL, plays a marginal role. What really matters to HD patients seems to be how the used CAM contributes to the perceived psychological support. Moreover, when we analyze the type of CAM used, we notice that the observed positive relationship between CAM use and HD patients’ psychological QoL is driven by two groups: patients that uses prayer-based practices and patients that practice meditation. Consistently with our interpretation, these are either spiritual or mind–body practices mainly used to find a general balance in life. This conclusion is in line with previous results suggesting that, for people suffering from ESRD, psychosocial factors are as important as their medical condition [67]. The use of CAM among HD patients seems to complete the role of official medicine under a holistic perspective rather than take its place.

## Conclusions

ESRD, like many chronic diseases, is a major concern worldwide as it requires healthcare professionals to focus their attention on patients’ QoL. The healthcare system can sometimes fail to address patients’ concerns about their QoL so the use of CAM is presented as a way to fill this void: patients’ use of CAM to compensate the negative consequences of the symptoms they have [37, 68]. Our results confirm this idea but suggest a more articulated interpretation. The use of CAM in our sample of HD patients is, indeed, largely present but it seems to be connected only to the psychological dimension of patients’ QoL, not to both the psychological and physical dimensions of QoL, as we would expect to see if CAM were used to directly relieve symptoms negative consequences. CAM seems to be used not as a substitute of official medicine but as a parallel support to HD patients’ QoL. Consistent with this interpretation, we observed that two groups of CAM users are most clearly connected with HD patients’ psychological QoL: the users of prayer-based practices and users of meditation. These are either spiritual or mind–body practices that have a larger scope than just to cope with the consequences of specific health conditions.

Further research is needed, in particular longitudinal and experimental studies analyzing the direct effects of CAM use on the different aspects of HD patients QoL. Nevertheless, our study shows that CAM use among HD patients may have a supporting role for patients’ QoL that isn’t strictly connected with their experience of symptoms or their health condition. Consequently, when framing CAM use among HD patients, healthcare professionals must consider the holistic role of these practices in the life of HD patients and not simply focus on the effects they have on their physical condition.

## Supplementary Information

**Additional file 1.**

## Data Availability

Due to privacy regulations, the original database cannot be provided except for quality control reasons. Analysis scripts are available under request. In both cases, all requested can be sent to the corresponding author.

## Abbreviations

ESRD: End Stage Renal Disease
HD: Hemodialysis
QoL: Quality of Life
CAM: Complementary and Alternative Medicines
DSI: Dialysis Symptom Index
WHOQOL-BREF: World Health Organization Quality of Life Bref
I-CAM-Q: International-Complementary and Alternative Médicine-Questionnaire

## Footnotes

1 The absence of relationship between CAM use and “overall QoL” and two dimensions “psychological QoL” and “physical QoL” was tested and confirmed.
