# Supplementary material for: Relationship among symptoms, quality of life, and complementary and alternative medicines use among patients undergoing hemodialysis in French-speaking Switzerland
Source: BMC Complement Med Ther. 2023 May 31;23:174. doi: 10.1186/s12906-023-04001-z (PMC10230675; doi:10.1186/s12906-023-04001-z)
Supplement: Supplementary file 1 — Additional file 1. [file 12906_2023_4001_MOESM1_ESM.pdf]

Section 1 – sociodemographic data

(Authors’ translation from French)

Identification code :____________

Date :_________

1) What's your age ? _________

   ☐ I refuse to answer

2) What's your sex ?
   ☐ Male
   ☐ Female

   ☐ I refuse to answer

3) What's your civil status?
   ☐ Single
   ☐ Married
   ☐ Widow
   ☐ Separated
   ☐ Living in a free union

   ☐ I refuse to answer

4) What's your activity status?
   ☐ Active work percentage: ____ %
   ☐ Retired
   ☐ Unemployed
   ☐ Other inactive

   ☐ I refuse to answer

5) Do you have any child?
   ☐ Yes
   ☐ No

   ☐ I refuse to answer

   If yes, how many are currently leaving with you?

   ____

   ☐ I refuse to answer

6) Are you a smoker?
   ☐ Non-smoker
   ☐ Former smoker
   ☐ Current smoker

   ☐ I refuse to answer

7) How long have you been on dialysis ?

   _____ months

   ☐ I don't know ☐ I refuse to answer

8) Have you ever used another method of dialysis?
   ☐ Yes
   ☐ No

   ☐ I don't know ☐ I refuse to answer

9) If yes, which type of dialysis have you used?
   ☐ Peritoneal dialysis
   ☐ Home Hemodialysis

   ☐ I don't know ☐ I refuse to answer

10) Would you like to have a renal transplantation in the future?

☐ Yes
☐ No
☐ I don't know ☐ I refuse to answer

11) Are you assuming any of the following product (multiple answers are possible)?

☐ Antihypertensive drugs
☐ Vitamin supplements
☐ Iron supplements
☐ Pain killers
☐ Antidepressants
☐ Other
☐ I don't know ☐ I refuse to answer

12) Are you currently suffering from one of these conditions?

Heart insufficiency
☐ Yes
☐ No
☐ I don't know ☐ I refuse to answer

Hypertension
☐ Yes
☐ No
☐ I don't know ☐ I refuse to answer

Diabetes
☐ Yes
☐ No
☐ I don't know ☐ I refuse to answer

Chronic obstructive pulmonary disease (COPD)
☐ Yes
☐ No
☐ I don't know ☐ I refuse to answer

Arrhythmia
☐ Yes
☐ No
☐ I don't know ☐ I refuse to answer

Lower limbs arteritis
☐ Yes
☐ No
☐ I don't know ☐ I refuse to answer

Cancer
☐ Yes
☐ No
☐ I don't know ☐ I refuse to answer

Hepatitis
☐ Yes
☐ No
☐ I don't know ☐ I refuse to answer

Section 2 – dialysis symptom index

(from :
Weisbord, S. D., Fried, L. F., Arnold, R. M., Rotondi, A. J., Fine, M. J., Levenson, D. J., & Switzer, G. E. (2004). Development of a symptom assessment instrument for chronic hemodialysis patients: the Dialysis Symptom Index. Journal of pain and symptom management, 27(3), 226–240. https://doi.org/10.1016/j.jpainsymman.2003.07.004)

Appendix

Dialysis Symptom Index

Instructions

Below is a list of physical and emotional symptoms that people on dialysis may have. For each symptom, please indicate if you had the symptom during the past week by circling "yes" or "no." If "yes," please indicate how much that symptom bothered you by circling the appropriate number.

During the past week: Did you experience this symptom?		*If "yes": How much did it bother you?* **Not At All**	**A Little Bit**	**Some-what**	**Quite a Bit**	**Very Much**
1. Constipation	NO					
	YES →	0	1	2	3	4
2. Nausea	NO					
	YES →	0	1	2	3	4
3. Vomiting	NO					
	YES →	0	1	2	3	4
4. Diarrhea	NO					
	YES →	0	1	2	3	4
5. Decreased appetite	NO					
	YES →	0	1	2	3	4
6. Muscle cramps	NO					
	YES →	0	1	2	3	4
7. Swelling in legs	NO					
	YES →	0	1	2	3	4
8. Shortness of breath	NO					
	YES →	0	1	2	3	4
9. Lightheadedness or dizziness	NO					
	YES →	0	1	2	3	4

During the past week: Did you experience this symptom?		*If "yes": How much did it bother you?* **Not At All**	**A Little Bit**	**Some-what**	**Quite a Bit**	**Very Much**
10. Restless legs or difficulty keeping legs still	NO<br>YES →	0	1	2	3	4
11. Numbness or tingling in feet	NO<br>YES →	0	1	2	3	4
12. Feeling tired or lack of energy	NO<br>YES →	0	1	2	3	4
13. Cough	NO<br>YES →	0	1	2	3	4
14. Dry mouth	NO<br>YES →	0	1	2	3	4
15. Bone or joint pain	NO<br>YES →	0	1	2	3	4
16. Chest pain	NO<br>YES →	0	1	2	3	4
17. Headache	NO<br>YES →	0	1	2	3	4
18. Muscle soreness	NO<br>YES →	0	1	2	3	4
19. Difficulty concentrating	NO<br>YES →	0	1	2	3	4
20. Dry skin	NO<br>YES →	0	1	2	3	4
21. Itching	NO<br>YES →	0	1	2	3	4
22. Worrying	NO<br>YES →	0	1	2	3	4

During the past week: Did you experience this symptom?		*If "yes": How much did it bother you?*				
		**Not At All**	**A Little Bit**	**Some -what**	**Quite a Bit**	**Very Much**
23. Feeling nervous	NO					
	YES →	0	1	2	3	4
24. Trouble falling asleep	NO					
	YES →	0	1	2	3	4
25. Trouble staying asleep	NO					
	YES →	0	1	2	3	4
26. Feeling irritable	NO					
	YES →	0	1	2	3	4
27. Feeling sad	NO					
	YES →	0	1	2	3	4
28. Feeling anxious	NO					
	YES →	0	1	2	3	4
29. Decreased interest in sex	NO					
	YES →	0	1	2	3	4
30. Difficulty becoming sexually aroused	NO					
	YES →	0	1	2	3	4

Are there any other symptoms not mentioned on this questionnaire that you have experienced during the past week? ____________________

The University of Pittsburgh Medical Center

VA Pittsburgh Healthcare System

Section 3 – WHOQOL-BREF

(from :
https://www.who.int/tools/whoqol/whoqol-bref)

Appendix III: Australian WHOQOL-BREF

WORLD HEALTH ORGANISATION QUALITY OF LIFE

WHOQOL- BREF

Australian Version (May 2000)

Instructions:

This assessment asks how you feel about your quality of life, health, and other areas of your life. Please answer all the questions. If you are unsure about which response to give to a question, please choose the one that appears most appropriate. This can often be your first response.

Please keep in mind your standards, hopes, pleasures and concerns. We ask that you think about your life in the **last two weeks**.

**Do you get the kind of support from others that you need?**				
Not at all<br>1	Slightly<br>2	Moderately<br>3	Very<br>④	Completely<br>5

You would circle the number 4 if in the last two weeks you got a great deal of support from others.

If you did not get any of the support from others that you needed in the last two weeks you would circle 1.

Thank you for your help.

Now turn to the back of this page

Please read the question, assess your feelings, for the last two weeks, and circle the number on the scale for each question that gives the best answer for you.

		Very poor	Poor	Neither poor nor good	Good	Very good
1	How would you rate your quality of life?	1	2	3	4	5

		Very dissatisfied	Fairly Dissatisfied	Neither satisfied nor dissatisfied	Satisfied	Very satisfied
2	How satisfied are you with your health?	1	2	3	4	5

The following questions ask about how much you have experienced certain things in the **last two weeks**.

		Not at all	A Small amount	A Moderate amount	A great deal	An Extreme amount
3	To what extent do you feel that physical pain prevents you from doing what you need to do?	1	2	3	4	5
4	How much do you need any medical treatment to function in your daily life?	1	2	3	4	5
5	How much do you enjoy life?	1	2	3	4	5
6	To what extent do you feel your life to be meaningful?	1	2	3	4	5

		Not at all	Slightly	Moderately	Very	Extremely
7	How well are you able to concentrate?	1	2	3	4	5
8	How safe do you feel in your daily life?	1	2	3	4	5
9	How healthy is your physical environment?	1	2	3	4	5

		Not at all	Slightly	Somewhat	To a great extent	Completely
10	Do you have enough energy for everyday life?	1	2	3	4	5
11	Are you able to accept your bodily appearance?	1	2	3	4	5
12	Have you enough money to meet your needs?	1	2	3	4	5
13	How available to you is the information you need in your daily life?	1	2	3	4	5
14	To what extent do you have the opportunity for leisure activities?	1	2	3	4	5

		Not at all	Slightly	Moderately	Very	Extremely
15	How well are you able to get around physically?	1	2	3	4	5

The following questions ask you to say how good or satisfied you have felt about various aspects of your life over the **last two weeks**.

		Very Dissatisfied	Fairly Dissatisfied	Neither Satified nor Dissatisfied	Satisfied	Very satisfied
16	How satisfied are you with your sleep?	1	2	3	4	5
17	How satisfied are you with your ability to perform your daily living activities?	1	2	3	4	5
18	How satisfied are you with your capacity for work	1	2	3	4	5
19	How satisfied are you with yourself?	1	2	3	4	5
20	How satisfied are you with your personal relationships?	1	2	3	4	5

21	How satisfied are you with your sex life?	1	2	3	4	5
22	How satisfied are you with the support you get from your friends?	1	2	3	4	5
23	How satisfied are you with the conditions of your living place?	1	2	3	4	5
24	How satisfied are you with your access to health services?	1	2	3	4	5
25	How satisfied are you with your transport?	1	2	3	4	5

The following question refers to **how often** you have felt or experienced certain things in the last two weeks.

		Never	Infrequently	Sometimes	Frequently	Always
26	How often do you have negative feelings such as blue mood, despair, anxiety or depression?	1	2	3	4	5

THE END

This translation was not created by the World Health Organization (WHO). WHO is not responsible for the content or accuracy of this translation. In the event of any inconsistency between the English and the translated version, the original English version shall be the binding and authentic version.

Section 4 – I-CAM-Q

(from :
Quandt, S. A., Verhoef, M. J., Arcury, T. A., Lewith, G. T., Steinsbekk, A., Kristoffersen, A. E., Wahner-Roedler, D. L., & Førnebø, V. (2009). Development of an international questionnaire to measure use of complementary and alternative medicine (I-CAM-Q). Journal of alternative and complementary medicine (New York, N.Y.), 15(4), 331–339. https://doi.org/10.1089/acm.2008.0521)

Appendix

NAFKAM International CAM Questionnaire (I-CAM-Q): RECOMMENDED FOR USE IN STUDIES OF COMPLEMENTARY AND ALTERNATIVE MEDICINE (CAM) -- Self-Administered Version

1. ***Visiting health care providers:*** Health problems may be attended to by a variety of complementary and conventional health care providers.

Have you seen any of the following providers in the last 12 months?	Yes	No	Number of times you saw this provider in the last 3 months?	Please indicate the *main* reason you *last* saw the provider (Check only *one*). For an acute illness/condition, one that lasted less than one month	To treat a long-term health condition (one that lasted more than one month) or its symptoms	To improve well-being	Other (Please specify the other reason)	How helpful was it for you to see this provider? (Check only one) Very	Somewhat	Not at all	Don't know
**Physician**	☐	☐	___	☐	☐	☐	___	☐	☐	☐	☐
**Chiropractor**	☐	☐	___	☐	☐	☐	___	☐	☐	☐	☐
**Homeopath**	☐	☐	___	☐	☐	☐	___	☐	☐	☐	☐
**Acupuncturist**	☐	☐	___	☐	☐	☐	___	☐	☐	☐	☐
**Herbalist**	☐	☐	___	☐	☐	☐	___	☐	☐	☐	☐
**Spiritual healer**	☐	☐	___	☐	☐	☐	___	☐	☐	☐	☐
**Specified option:** ___	☐	☐	___	☐	☐	☐	___	☐	☐	☐	☐
**Other (please specify):** ___	☐	☐	___	☐	☐	☐	___	☐	☐	☐	☐
**Other (please specify):** ___	☐	☐	___	☐	☐	☐	___	☐	☐	☐	☐

2. Complementary treatments received from physicians (MDs)

If you have not seen a physician in the past 12 months, please go to question 3.
Some physicians provide complementary, as well as conventional treatments

Have you received any of the following complementary treatments from a physician in the last 12 months?	Yes	No	Number of times you received this treatment in the last 3 months?	Please indicate the *main* reason you *last* received this treatment (Check only *one*). For an acute illness/condition, one that lasted less than one month	To treat a long-term health condition (one that lasted more than one month) or its symptoms	To improve well-being	Other (Please specify the other reason)	How helpful was it to receive treatment from the physician? (Check only one) Very	Somewhat	Not at all	Don't know
**Manipulation**	☐	☐	____	☐	☐	☐	____	☐	☐	☐	☐
**Homeopathy**	☐	☐	____	☐	☐	☐	____	☐	☐	☐	☐
**Acupuncture**	☐	☐	____	☐	☐	☐	____	☐	☐	☐	☐
**Herbs**	☐	☐	____	☐	☐	☐	____	☐	☐	☐	☐
**Spiritual healing**	☐	☐	____	☐	☐	☐	____	☐	☐	☐	☐
**Specified option:** ____	☐	☐	____	☐	☐	☐	____	☐	☐	☐	☐
**Other (please specify):** ____	☐	☐	____	☐	☐	☐	____	☐	☐	☐	☐

3. Use of Herbal Medicine and Dietary Supplements, including tablets, capsules and liquids.

For each category below, please list up to three products you have used in the last 12 months.	Do you <u>currently</u> use this product?		Please indicate the *main* reason that applies to your *last* use (Check only *one*).				How helpful did you find this product? (Check only one)			
	Yes	No	For an acute illness/condition, one that lasted less than one month	To treat a long-term health condition (one that lasted more than one month) or its symptoms	To improve well-being	Other (Please specify)	Very	Somewhat	Not at all	Don't know
**Herbs/Herbal Medicine**										
____________	☐	☐	☐	☐	☐	____________	☐	☐	☐	☐
____________	☐	☐	☐	☐	☐	____________	☐	☐	☐	☐
____________	☐	☐	☐	☐	☐	____________	☐	☐	☐	☐
**Vitamins/Minerals**										
____________	☐	☐	☐	☐	☐	____________	☐	☐	☐	☐
____________	☐	☐	☐	☐	☐	____________	☐	☐	☐	☐
____________	☐	☐	☐	☐	☐	____________	☐	☐	☐	☐
**Homeopathic remedies**										
____________	☐	☐	☐	☐	☐	____________	☐	☐	☐	☐
____________	☐	☐	☐	☐	☐	____________	☐	☐	☐	☐
____________	☐	☐	☐	☐	☐	____________	☐	☐	☐	☐
**Other Supplements**										
____________	☐	☐	☐	☐	☐	____________	☐	☐	☐	☐
____________	☐	☐	☐	☐	☐	____________	☐	☐	☐	☐
____________	☐	☐	☐	☐	☐	____________	☐	☐	☐	☐

4. Self Help Practices

Have you used any of the following self-help practices in the last 12 months?	Yes	No	Number of times you used this practice in the last 3 months?	Please indicate the *main* reason that applies to your *last* use of the self-help practice (Check only *one*).				How helpful did you find this self-help practice? (Check only one)			
				For an acute illness/condition, one that lasted less than one month	To treat a long-term health condition (one that lasted more than one month) or its symptoms	To improve well-being	Other (Please specify the other reason)	Very	Somewhat	Not at all	Don't know
**Meditation**	☐	☐	____	☐	☐	☐	____________	☐	☐	☐	☐
**Yoga**	☐	☐	____	☐	☐	☐	____________	☐	☐	☐	☐
**Qigong**	☐	☐	____	☐	☐	☐	____________	☐	☐	☐	☐
**Tai Chi**	☐	☐	____	☐	☐	☐	____________	☐	☐	☐	☐
**Relaxation techniques**	☐	☐	____	☐	☐	☐	____________	☐	☐	☐	☐
**Visualization**	☐	☐	____	☐	☐	☐	____________	☐	☐	☐	☐
**Attended traditional healing ceremony**	☐	☐	____	☐	☐	☐	____________	☐	☐	☐	☐
**Praying for own health**	☐	☐	____	☐	☐	☐	____________	☐	☐	☐	☐
**Specified option:** ____________	☐	☐	____	☐	☐	☐	____________	☐	☐	☐	☐
**Other (please specify):** ____________	☐	☐	____	☐	☐	☐	____________	☐	☐	☐	☐

Section 5 – Biological values

(Authors' translation from French)

Identification code :______________
Date :______________

We would like to collect some biological data from your medical records, would you agree?
☐ Agree
☐ Refuse

Clinical data from medical records:

Protein concentration : ____________
Hemoglobin (g/dl) : ____________
Creatine : ____________
Glomerular filtration rate : ____________

Weight : ____________ kg
Size : ____________ cm
